# Nomograms for Predicting the Risk of SIRS and Urosepsis after Uroscopic Minimally Invasive Lithotripsy

**DOI:** 10.1155/2022/6808239

**Published:** 2022-03-11

**Authors:** Can Wang, Rufu Xu, Yuanning Zhang, Yingbing Wu, Teng Zhang, Xingyou Dong, Rong Zhang, Xuelian Hu

**Affiliations:** ^1^Department of Pharmacy, The Second Affiliated Hospital of Army Medical University, Chongqing 400037, China; ^2^Department of Pharmacy, University Town Hospital Affiliated of Chongqing Medical University, Chongqing 401331, China; ^3^Department of Urology Surgery, The Second Affiliated Hospital of Army Medical University, Chongqing 400037, China

## Abstract

**Objective:**

To analyze the potential risk factors that affect the development of urosepsis following uroscopic minimally invasive lithotripsy and to develop a nomogram that predicts the probability of postoperative urosepsis.

**Methods:**

We retrospectively analyzed the clinical data from patients that underwent percutaneous nephrolithotripsy (PCNL) or ureteroscopic lithotripsy (URL) between January 2018 and December 2019. The enrolled patients were grouped twice according to systemic inflammatory response syndrome (SIRS) and quick sequential organ failure assessment (qSOFA). After univariate and multivariate logistic regression analyses, we identified the independent predictive factors affecting the development of postoperative SIRS and urosepsis, and built the nomograms.

**Results:**

From January 2018 to December 2019, 1959 patients underwent PCNL or URL, of whom 236 patients were accorded with the inclusion criteria. Of all 236 patients, 64 (27.12%) patients developed postoperative SIRS, and 17 (7.20%) patients developed postoperative urosepsis. Multivariate logistic regression analysis showed that positive preoperative urine culture (PUC+) (OR = 2.331, *P* = 0.044), procalcitonin (PCT) (OR = 1.093, *P* = 0.037), C-reactive protein (CRP) (OR = 1.017, *P* < 0.001), and neutrophil ratio (NEUT%) (OR = 1.091, *P* = 0.004) of postoperative were independent predictors of SIRS, and PCT (OR = 1.017, *P* = 0.003) and CRP (OR = 1.080, *P* < 0.001) were independent predictors of urosepsis. Additionally, the nomograms demonstrated good accuracy in predicting SIRS and urosepsis with a C-index of 0.884 (95% CI: 0.835-0.934) and 0.941 (95% CI: 0.885-0.996), respectively.

**Conclusions:**

The nomograms achieved the prediction of SIRS and urosepsis after uroscopic minimally invasive lithotripsy. Using this model, the risk of SIRS or urosepsis for an individual patient can be determined, which facilitates early diagnosis and rational treatment.

## 1. Introduction

Urolithiasis is one of the most common urologic diseases with increasing prevalence each year around the world. The prevalence of urolithiasis was 8.8% in North America [1] and 6.5% in China [2]. The management of urinary tract stones has evolved from traditional open surgeries to minimally invasive endourological procedures, among which percutaneous nephrolithotripsy (PCNL) and ureteroscopic lithotripsy (URL) have become the preferred treatment options for patients with upper urinary tract stones [3]. Compared with traditional open surgery, uroscopic minimally invasive lithotripsy is safer and more efficient, but postoperative fever, urinary tract infection, bleeding, and other complications are still very common [4].

About 21.0-32.1% of the patients experienced infection symptoms such as fever after PCNL, and 0.3-4.7% of the patients developed urosepsis [5]. Urosepsis may rapidly progress to septic shock with a mortality as high as 20–42% if patients are not diagnosed in time [6, 7]. Therefore, we can minimize the adverse consequences of urosepsis if we can effectively predict the risk of urosepsis and timely treat. Biomarker detection is rapid and has certain significance for the early diagnosis of sepsis. Previous studies [8, 9] found that the main risk factors were preoperative and intraoperative factors, including positive urine cultures, stone surgery history, stone size, stone complexity, and operation time. In a small number of studies [10, 11], risk factors included biomarkers, and these studies were limited to two or three biomarkers, which demand further research and exploration. Therefore, the objective of this study was to evaluate the risk factors of SIRS and urosepsis after uroscopic minimally invasive lithotripsy and to establish nomogram prediction models for predicting the probability of postoperative SIRS and urosepsis.

## 2. Patients and Methods

### 2.1. Patients

Between January 2018 and December 2019, patients who underwent uroscopic minimally invasive lithotripsy in the Second Affiliated Hospital of Army Medical University were retrospectively analyzed. The exclusion criteria were (1) age < 18 years old, (2) accompanied by pneumonia or other parts of infection, (3) basic diseases of blood system diseases, (4) diseases of the immune system or undergoing immunomodulatory therapy, and (5) incomplete laboratory data. The study was approved by the Institutional Ethics Committee of the Second Affiliated Hospital of Army Medical University.

### 2.2. Group Standard

Patients were divided into two groups according to whether they had systemic inflammatory response syndrome (SIRS) or urosepsis after surgery. SIRS was defined as the occurrence of any 2 or more of the following 4 criteria: temperature > 38°C or <36°C; heart rate > 90/min; respiratory rate > 20/min or PaCO2 < 32 mmHg (4.3 kPa); white blood cell count > 12000/mm^3^ or <4000/mm^3^ or >10% immature bands [12]. Sepsis was defined as the presence of ≥2 quick Sequential Organ Failure Assessment (qSOFA) score in the following criteria: respiratory rate ≥ 22/min, altered mentation (Glasgow Coma Scale score < 13), and systolic blood pressure ≤ 100 mmHg [13].

### 2.3. Risk Factors

We collected and analyzed the following factors for the subjects: general information, surgical information, preoperative examination, and postoperative examination (Table 1). The diameter of calculi was measured by CT. To minimize bias, the most abnormal physiological and laboratory values were recorded.

### 2.4. Statistical Analysis

The continuous variables of normal distribution were expressed as mean ± SD and compared using Student's *t*-test, while the nonnormal distribution was expressed by median (IQR) and compared using the Mann–Whitney test. Categorical variables were compared using the *χ*^2^ test or Fisher exact test. Univariate and multivariate logistic regression analyses were used to analyze the risk factors, and the risk factors with *P* < 0.1 in the univariate logistic regression analysis were included in multivariate logistic regression analysis. A nomogram was formulated and verified based on the results from multivariate logistic regression analysis using R statistical software. The prediction performance of the nomogram was measured by consistency index (C-index) and calibration of bootstrap samples to reduce the over-fitting deviation. In all analyses, *P* < 0.05 was considered to indicate statistical significance.

## 3. Results

### 3.1. Participant Characteristics

During the study period, 1959 consecutive patients underwent uroscopic minimally invasive lithotripsy. Of these, 236 patients met the inclusion criteria and were enrolled. Of all 236 patients, 64 (27.12%) patients developed postoperative SIRS, and 17 (7.20%) patients developed postoperative urosepsis. The patient demographics were described in Table 1.

### 3.2. Independent Risk Factors

The results of univariate and multivariate logistic analysis for SIRS are presented in Table 2. In the univariate analysis, there were significant differences between groups with SIRS and non-SIRS in positive preoperative urine culture (PUC+), positive urine white blood cell (urine WBC+), positive urine nitrite (NIT+), urine bacterial count, Cystain C (Cys-C), Glomerular Filtration Rate (GFR), procalcitonin (PCT), C-reactive protein (CRP), D-dimer, WBC, neutrophil ratio (NEUT%), platelet (PLT), and eosinophil ratio (EO%). In the multivariate analysis, PUC+ (OR = 2.331; 95% CI: 1.022-5.317), PCT (OR = 1.093; 95% CI: 1.005-1.187), CRP (OR = 1.017; 95% CI: 1.009-1.024), and NEUT% (OR = 1.091; 95% CI: 1.029-1.157) were independently associated with SIRS.

The results of univariate and multivariate logistic analysis for urosepsis are presented in Table 3. In the univariate analysis, there were significant differences between groups with urosepsis and nonurosepsis in history of urolithiasis surgery, PUC+, urine WBC+, NIT+, urine bacterial count, Cys-C, GFR, PCT, CRP, D2-F, WBC, NEUT%, and PLT. In the multivariate analysis, PCT (OR = 1.017; 95% CI: 1.006-1.029) and CRP (OR = 1.080; 95% CI: 1.042-1.120) were independently associated with urosepsis.

### 3.3. Development and Validation of Nomograms

**Figure 1:**
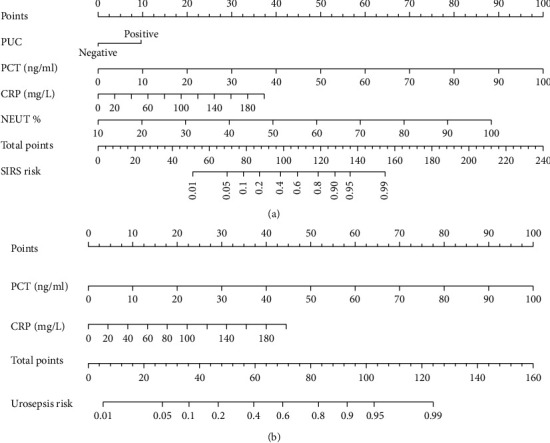
Nomograms for SIRS and urosepsis in the patient with uroscopic minimally invasive lithotripsy. (a) Nomogram to estimate the risk of SIRS. PUC: preoperative urine cultures. (b) Nomogram to estimate the risk of urosepsis.

**Figure 2:**
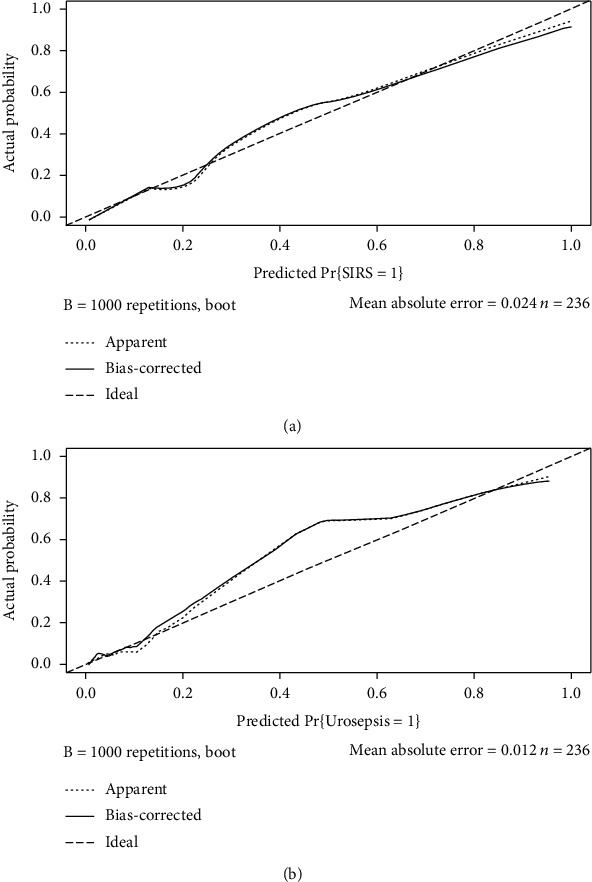
Calibration curve of the prediction model for SIRS and urosepsis in the patient with uroscopic minimally invasive lithotripsy. (a) Calibration curve of the prediction model for SIRS. (b) Calibration curve of the prediction model for urosepsis.

These independently associated risk factors were used to form SIRS and urosepsis risk estimation nomogram (Figure 1). The sum of points in the nomogram demonstrated the probability of SIRS or urosepsis (the bottom scales). The two nomograms were internally validated by computing the bootstrap-corrected C-index and using the calibration plot. The nomogram demonstrated good accuracy in estimating the risk of SIRS with an unadjusted C index of 0.884 (95% CI: 0.835-0.934) and a bootstrap-correct C index of 0.877. The nomogram also demonstrated great accuracy in estimating the risk of urosepsis with an unadjusted C index of 0.941 (95% CI: 0.885-0.996) and a bootstrap-correct C index of 0.939. In addition, calibration curves of nomograms showed that the SIRS and urosepsis probabilities predicted by the nomograms agreed well with the actual probabilities (Figure 2).

## 4. Discussion

With the advancement of medical devices and technology, uroscopic minimally invasive lithotripsy has been developed. However, compared with other operations, this operation is more prone to postoperative infection. There are a large number of colonized bacteria and endotoxins on most calculi [14]. The stones can lead to partial or total obstruction of the urinary system before the operation, forming a microenvironment where bacteria can grow easily [15]. In addition, a large amount of normal saline is needed for high-pressure perfusion during the operation to maintain a clear visual field. With the increase of pressure, the perfusion fluid can flow back through various renal pelvic veins, renal pelvic tubules, renal pelvic lymph, and other channels, so that bacteria and endotoxin enter the blood with urine [16]. Our study shows the incidence of postoperative SIRS and sepsis was 3.27% and 0.87%, which is similar to that found by previous studies [5]. The delay of diagnosis is one of the main reasons for the high mortality of urosepsis. The mortality of sepsis may increase by 8% for every hour delay of diagnosis [17]. At present, the sepsis definition has been updated to sepsis-3, which uses the standard score of SOFA or qSOFA. However, SOFA is not only time-consuming but also expensive for frequent laboratory tests, and it is usually only applicable in ICU. Although the qSOFA is simpler and faster than SOFA, it has low sensitivity in diagnosing sepsis. Urosepsis develops rapidly after endoscopic lithotripsy; therefore, a highly sensitive diagnostic method is needed. The pathogenesis of sepsis is mainly cytokine storm caused by immune response disorder, and the occurrence of SIRS is considered as the first step of sepsis [18]. A large meta-analysis showed that the sensitivity of SIRS was significantly better than that of qSOFA in the diagnosis of sepsis [19]. Therefore, the identification of SIRS has a certain clinical significance for the early diagnosis of sepsis. Although SIRS has high sensitivity, it has low specificity, which not only leads to a substantial increase in medical costs but also the excessive use of high-grade antibiotics increases drug resistance [20]. Therefore, there is still a dispute about the diagnostic criteria of sepsis. We established the prediction models of SIRS and urosepsis after uroscopic minimally invasive lithotripsy.

In this study, positive preoperative urine culture was identified as an independent risk factor for SIRS. Currently, there remains controversy regarding the predicting value of preoperative urine culture for urosepsis after uroscopic minimally invasive lithotripsy. Some studies believed that preoperative urine culture has predictive value [9, 18], while some other studies reported that the results of preoperative middle urine culture are not accurate enough to have predictive value, due to urinary tract obstruction caused by calculi [21, 22]. In our study, the positive preoperative urine culture has a certain predictive value for urosepsis, and the results have guiding significance for the use of perioperative antibiotics and the treatment of postoperative infection.

The detection of biomarkers is rapid, and some biomarkers have clinical significance in the early diagnosis of urosepsis [23]. In the present study, PCT and CRP were identified as independent risk factors of SIRS and urosepsis. NEUT% is shown to be an independent risk factor for SIRS, but not for urosepsis. The reason may be that the degree of infection is generally proportional to the number of neutrophils, but the number of neutrophils may decrease in severe infection. PCT, the inactive pro-peptide of calcitonin, is a precursor protein with no hormonal activity and has the characteristics of wide biological range, short induction time after bacterial stimulation, and long half-life [24]. CRP is an acute inflammatory protein that increases up to 1,000-fold at sites of infection or inflammation [25]. A meta-analysis [26] of PCT and CRP as diagnostic markers of sepsis showed that the sensitivity and specificity of PCT were 0.77 and 0.79, respectively, and the sensitivity and specificity of CRP were 0.73 and 0.61, respectively. The results showed that the specificity of CRP was significantly lower than that of PCT because the increase of CRP was affected by many other factors, such as rheumatologic diseases, malignancy, and drug reactions [27]. Both PCT and CRP have moderate diagnostic value for sepsis, but it is still limited to diagnose sepsis as a single index. Our study confirmed that the combination of PCT and CRP has a good predictive value for urosepsis. In addition, our study showed that 88.2% of urosepsis the patients developed occurred within 24 hours after uroscopic minimally invasive lithotripsy. Therefore, it is recommended to detect PCT and CRP within 24 hours after operation.

Although the above risk factors are independent of each other, they also have a certain correlation, which may affect each other clinically. Therefore, we build two nomograms according to the relative risk of each factor, which is convenient for clinical application. In clinical application, physicians can use the nomograms to predict the corresponding score of risk factors, and the corresponding postoperative SIRS and urosepsis risk index can be obtained. Physicians can judge the severity of the disease by nomogram to choose the appropriate antibiotics and treatment. For example, the patient with high risk of sepsis should be treated early according to the sepsis management, including administering one or more broad-spectrum antibiotics, hemodynamic monitoring, and fluid resuscitation when needed. The patient with high-risk of SIRS and low-risk of urosepsis need treatment of postoperative infection but does not need to be treated as sepsis.

There are some limitations in this study. The main limitations are the retrospective study and the limited number of patients. Therefore, more prospective studies with large sample are needed to validate the prediction effectiveness of the nomograms.

## 5. Conclusions

PUC+, PCT, CRP, and NEUT% are the risk factors of SIRS after uroscopic minimally invasive lithotripsy. PCT and CRP are risk factors of urosepsis after uroscopic minimally invasive lithotripsy. By combining risk factors, nomograms were constructed for SIRS and urosepsis. The models provide an early prediction method for SIRS and urosepsis after uroscopic minimally invasive lithotripsy, which facilitates early diagnosis and rational treatment.

## Figures and Tables

**Table 1:** Participant characteristics.

Factors	SIRS			Urosepsis		
	SIRS (*n* = 64)	Non-SIRS (*n* = 172)	*P* value	Urosepsis (*n* = 17)	Nonurosepsis (*n* = 219)	*P* value
Age (years)	52.27 ± 13.04	50.75 ± 12.90	0.424	54 (48.5-56.5)	51 (43-60)	0.360
Gender (male/female)	38/26	101/71	0.928	8/9	131/88	0.303
BMI (kg/m^2^)	25.12 ± 3.26	24.66 ± 3.23	0.340	23.64 ± 3.00	24.88 ± 3.24	0.128
Previous surgery for calculi, *n* (%)	34 (53.12%)	67 (38.95%)	0.050	13 (76.47%)	88 (40.18%)	0.004∗
Type of operation PCNL/URL	40/24	120/52	0.288	10/7	150/69	0.411
Operative time ≥ 120 (min), *n* (%)	12 (18.75%)	29 (16.86%)	0.733	3 (17.65%)	38 (17.35%)	0.975
Diameter of calculi (cm)	1.50 (1.10-2.55)	1.50 (1.10-2.00)	0.627	1.50 (1.85-1.15)	1.50 (1.00-2.20)	0.960
CT attenuation value of calculi (Hu)	1208.16 ± 140.378	1168.20 ± 179.230	0.109	1171.41 ± 109.947	1179.63 ± 174.190	0.848
PUC+, *n* (%)	36 (56.25%)	31 (18.02%)	<0.001∗	13 (76.47%)	54 (24.66%)	<0.001∗
Positive urine WBC, *n* (%)	45 (70.31%)	87 (50.58%)	0.007∗	14 (82.35%)	176 (80.37%)	0.842
Positive urine nitrite, *n* (%)	21 (32.81%)	17 (9.88%)	<0.001∗	8 (47.06%)	30 (13.70%)	<0.001∗
Urine bacterial count > 100/*μl*, *n* (%)	27 (42.19%)	35 (20.35%)	<0.001∗	11 (65.71%)	51 (23.29%)	<0.001∗
Cys-C (mg/L)	1.23 (0.98-1.67)	1.04 (0.88-1.32)	<0.001∗	1.26 (1.02-2.18)	1.07 (0.90-1.36)	0.013∗
GFR (ml/min/1.73 m^2^)	78.50 (45.75-92.00)	84.00 (69.25-100.00)	0.011∗	77.00 (36.73-90.82)	81.00 (66.00-99.00)	0.070
ALB (g/L)	41.50 ± 4.92	42.13 ± 4.98	0.389	40.21 ± 5.31	42.09 ± 4.92	0.132
PCT (ng/ml)	2.60 (0.42-18.71)	0.06 (0.03-0.19)	<0.001∗	31.97 (85.15-7.92)	0.08 (0.04-0.45)	<0.001∗
CRP (mg/L)	78.70 (40.32-143.50)	22.15 (8.50-63.93)	<0.001∗	129.60 (63.60-165.80)	32.30 (11.70-80.10)	<0.001∗
D-dimer (mg/L)	0.43 (0.15-1.20)	0.24 (0.15-0.55)	0.025∗	0.78 (0.32-8.79)	0.26 (0.15-0.59)	0.002∗
WBC (10^9^/L)	12.36 (9.29-16.35)	10.28 (8.44-12.56)	<0.001∗	14.25 (8.90-23.41)	10.63 (8.58-13.16)	0.024∗
NEUT%	88.70 (85.50-93.28)	80.40 (74.72-85.40)	<0.001∗	91.70 (87.55-95.85)	82.20 (76.50-88.20)	<0.001∗
PLT (10^9^/L)	191.00 (141.25-254.75)	205.50 (168.00-258.50)	0.029∗	171.00 (119.50-227.00)	203.00 (165.00-285.00)	0.032∗
EO% < 0.4, *n* (%)	55 (85.94%)	119 (69.19%)	0.009∗	16 (94.12%)	158 (72.15%)	0.047∗

**Table 2:** Univariate and logistic multivariate regression analysis of SIRS.

Factors	Univariate analysis			Multivariate analysis		
	OR	95% CI	*P* value	OR	95% CI	*P* value
Age	1.009	0.987-1.032	0.423			
Gender (female)	0.928	0.543-1.745	0.928			
BMI	1.044	0.956-1.141	0.339			
Previous surgery for calculi	1.776	0.996-3.168	0.052			
Type of operation (PCNL)	0.722	0.396-1.318	0.289			
Operative time ≥ 120 min	1.138	0.541-2.394	0.734			
Diameter of calculi	1.117	0.836-1.493	0.453			
CT attenuation value of calculi	1.002	1.000-1.003	0.113			
PUC+	5.848	3.119-10.964	<0.001∗	2.331	1.022-5.317	0.044∗
Positive urine WBC	2.314	1.252-4.275	0.007∗			
Positive urine nitrite	4.453	2.160-9.177	<0.001∗			
Urine bacterial count > 100/*μ*l	2.856	1.537-5.308	0.001∗			
Cys-C	1.809	1.217-2.690	0.003∗			
GFR	0.985	0.975-0.995	0.004∗			
ALB	0.975	0.920-1.033	0.387			
PCT	1.235	1.127-1.353	<0.001∗	1.093	1.005-1.187	0.037∗
CRP	1.020	1.013-1.026	<0.001∗	1.017	1.009-1.024	<0.001∗
D-dimer	1.248	1.056-1.473	0.009∗			
WBC	1.160	1.081-1.244	<0.001∗			
NEUT%	1.159	1.102-1.220	<0.001∗	1.091	1.029-1.157	0.004∗
PLT	0.995	0.991-0.999	0.019∗			
EO% < 0.4	2.722	1.253-5.911	0.011∗			

**Table 3:** Univariate and logistic multivariate regression analysis of urosepsis.

Factors	Univariate analysis			Multivariate analysis		
	OR	95% CI	*P* value	OR	95% CI	*P* value
Age	1.019	0.981-1.059	0.328			
Gender (female)	1.675	0.622-4.507	0.307			
BMI	0.883	0.753-1.036	0.128			
Previous surgery for calculi	4.838	1.528-15.322	0.007∗			
Type of operation (PCNL)	0.657	0.240-1.799	0.414			
Operative time ≥ 120 min	1.021	0.280-3.727	0.975			
Diameter of calculi	0.829	0.487-1.412	0.491			
CT attenuation value of calculi	1.000	0.997-1.003	0.848			
PUC+	9.931	3.107-31.742	<0.001∗			
Positive urine WBC	3.994	1.116-14.294	0.033∗			
Positive urine nitrite	5.600	2.005-15.644	0.001∗			
Urine bacterial count > 100/*μ*l	6.039	2.128-17.136	0.001∗			
Cys-C	1.879	1.193-2.960	0.007∗			
GFR	0.982	0.967-0.998	0.030∗			
ALB	0.928	0.843-1.023	0.132			
PCT	1.092	1.048-1.139	<0.001∗	1.017	1.006-1.029	0.003∗
CRP	1.019	1.010-1.027	<0.001∗	1.080	1.042-1.120	<0.001∗
D-dimer	1.329	1.111-1.589	0.002∗			
WBC	1.182	1.081-1.293	<0.001∗			
NEUT%	1.240	1.118-1.377	<0.001∗			
PLT	0.990	0.981-0.998	0.020∗			
EO% < 0.4	6.177	0.802-47.591	0.080			

## Data Availability

Data and material are available upon request.

## Supplementary Materials

Supplementary Materials Supplementary Figure 1: examples for nomograms to predict the probability of SIRS or urosepsis in the patient with uroscopic minimally invasive lithotripsy.
