# Supplementary material for: Nomograms for Predicting the Risk of SIRS and Urosepsis after Uroscopic Minimally Invasive Lithotripsy
Source: Biomed Res Int. 2022 Mar 11;2022:6808239. doi: 10.1155/2022/6808239 (PMC8933078; doi:10.1155/2022/6808239)
Supplement: Supplementary Materials — Supplementary Figure 1: examples for nomograms to predict the probability of SIRS or urosepsis in the patient with uroscopic minimally invasive lithotripsy. [file 6808239.f1.docx]

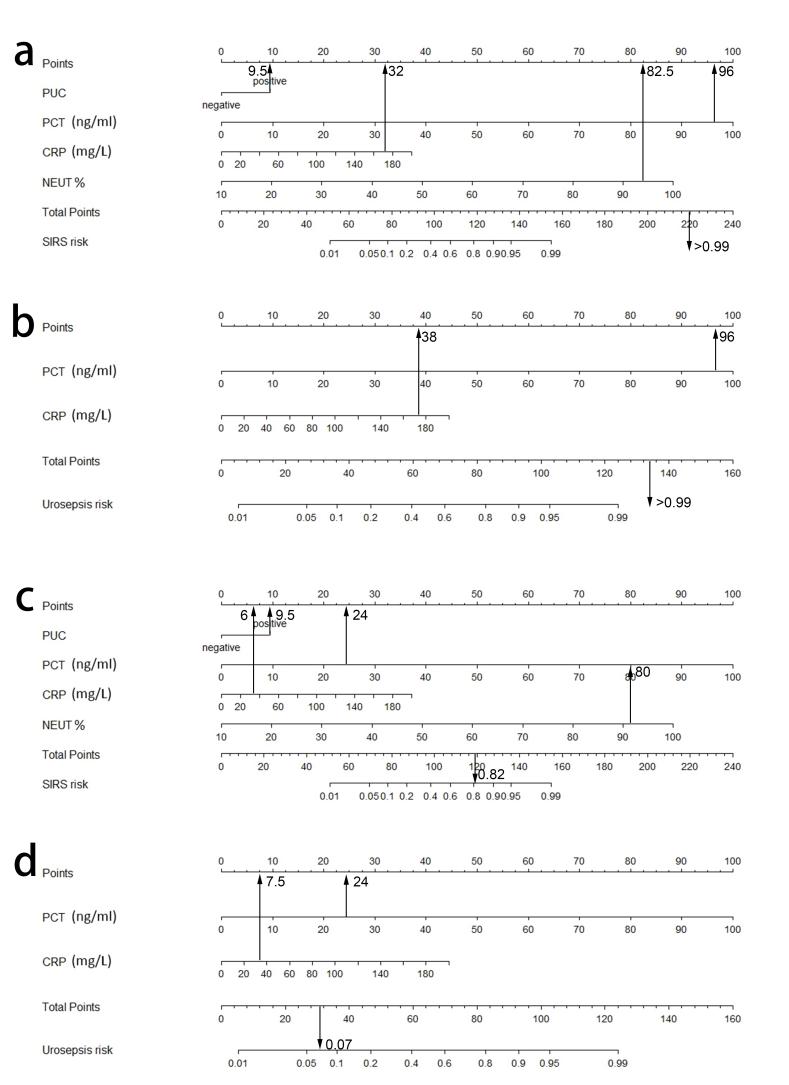


**Supplementary Figure 1:** Examples for nomograms to predict the probability of SIRS or urosepsis in the patient with uroscopic minimally invasive lithotripsy. a: Nomogram to estimate the risk of SIRS for patient 1. PUC, preoperative urine cultures; b: Nomogram to estimate the risk of urosepsis for patient 1. c: Nomogram to estimate the risk of SIRS for patient 2. PUC, preoperative urine cultures; d: Nomogram to estimate the risk of urosepsis for patient 2.

For example, patient 1: a middle-aged male patient after PCNL had PUC+, PCT=96 ng/ml, CRP=177 mg/L, and NEUT%=95%. In the SIRS risk estimate nomogram prediction model, the score of each prediction indicators were 9.5 points for PUC, 96 points for PCT, 32 points for CRP and 82.5 points for NEUT%, and the cumulative score was 9.5+96+ 32+ 82.5 =220 (**supplementary Fig. 1a**). In the nomogram predicts urosepsis, the cumulative score of each prediction indicators was 96 points for PCT and 38 points for CRP, and the cumulative score was 96+38= 134 (**supplementary Fig. 1b**). The corresponding predicted risk of SIRS and urosepsis were more than 0.99, so this patient has high-risk of SIRS and urosepsis. Patient 2: a middle-aged male patient after PCNL had PUC+, PCT=24 ng/ml, CRP=35 mg/L, and NEUT%=92%. In the nomogram predicts SIRS, the cumulative score of each prediction indicators was 9.5 +24 +6 +80 = 119.5, and the corresponding predicted risk of SIRS was 0.82 (**supplementary Fig. 1c**). In the nomogram predicts urosepsis, the cumulative score of each prediction indicators were 24 +7.5 = 31.5, and the corresponding predicted risk of urosepsis was 0.07 (**supplementary Fig. 1d**). According to the predicted probability above, this patient had high-risk of SIRS and low-risk of urosepsis.
